# Author’s Response to a Commentary on “Prevalence of Undiagnosed Hypertension Among Adult Displaced Individuals in Baidoa Camps, Somalia (Preprint)”

**DOI:** 10.2196/70265

**Published:** 2025-06-03

**Authors:** Mohamed Jayte

**Affiliations:** 1 Internal Medicine Department, Kampala International University, F46V+MW2, IshakaKampala, Uganda, 256 755272543

**Keywords:** prevalence, undiagnosed, epidemiology, heart, cardiology, cardiovascular, cross-sectional, survey, questionnaires, hypertension, blood pressure, poverty, sedentary, displaced, refugee, Africa

*This is the author’s response to a commentary on “Prevalence of Undiagnosed Hypertension Among Adult Displaced Individuals in Baidoa Camps, Somalia.”*

## Round 1 Review

### Anonymous [1]

*I commend the author for this study [2] on an important topic. However, here are a few comments to help improve the manuscript.*

#### Title

*1. The title needs some slight changes to improve clarity. For instance, what do you mean by “displaced individuals”? Would you rather state it as “internally displaced persons” or just “adults in Baidoa displacement camps”?*

**Response**: We have revised the title to clarify our subject. The new title now reads “[Revised Title: Internally Displaced Persons in Baidoa Displacement Camps].”

*2. Use a uniform font for the title.*

**Response**: The title font has been standardized to ensure uniformity throughout the manuscript.

#### Introduction

*1. Ensure a consistent referencing style throughout the manuscript.*

**Response**: We have revised the manuscript to ensure consistent referencing style throughout.

*2. In the sentence “Over the past few decades, the...,” delete the bracket at the end of the statement.*

**Response**: The bracket has been removed from the specified sentence.

*3. Check the overall grammar of the text throughout the manuscript.*

**Response**: We have conducted a thorough grammar check and corrected any errors found throughout the manuscript.

*4. Regarding the burden of hypertension, provide more updated statistics on hypertension, using both global and regional data. Ensure a clear linkage and transition between the two because, as it stands right now, the statistics are scattered throughout the introduction, rendering it redundant.*

**Response**: We have included updated global and regional statistics on hypertension and improved the linkage and transition between the two sets of data for better coherence.

*5. Provide more context on the displaced populations and their specific vulnerabilities to hypertension to strengthen the rationale of the study. Discuss the factors therein.*

**Response**: Additional context on the vulnerabilities of displaced populations to hypertension has been included, with a discussion of relevant factors contributing to this vulnerability.

*6. The section would benefit from a discussion on the effects of hypertension.*

**Response**: A new paragraph discussing the effects of hypertension has been added to the introduction.

*7. Cite studies that have investigated hypertension among displaced populations, if any exist, or state the deficit if none.*

**Response**: We have cited relevant studies investigating hypertension among displaced populations. Where studies are lacking, we have noted this deficit to highlight the gap in the literature.

*8. Discuss any interventions and strategies that have been implemented to tackle the problem of hypertension in these communities and state the possible gaps before your objective.*

**Response**: A discussion on interventions and strategies addressing hypertension in displaced communities has been added, including a mention of the existing gaps.

#### Methods

1. Formatting issue: provide a heading for your Methods section.

**Response**: A clear heading for the Methods section has been added.

2. As stated above, there is a need to improve the overall grammar.

**Response**: We have reviewed and improved the grammar throughout the Methods section.

*3. Provide more detail regarding the inclusion criteria. For instance, was there a specific displacement duration that was considered (ie, the minimum amount of time spent in the camp so far)?*

**Response:** We have specified the inclusion criteria, including the consideration of the duration of displacement in the camps.

*4. Provide a justification for the exclusion criteria*.

**Response:** A justification for the exclusion criteria has been included to clarify the rationale behind them.

*5. Provide the reference for “The sample size for this study was determined....”*

**Response**: The appropriate reference for the sample size determination has been added to the Methods section.

*6. Add more detail regarding the validation of the questionnaire. Was it adopted from previous studies? Was it pretested?*

**Response:** We have provided additional details about the validation of the questionnaire, including its adoption from previous studies and the pretesting process.

*7. Add detail on the measurement of blood pressure (BP). Who measured the BPs? Were they trained? How did you deal with white-coat hypertension? What was the interval between the different BP readings?*

**Response:** We have added detailed information on blood pressure measurement, including who conducted the measurements, their training, how white-coat hypertension was addressed, and the intervals between readings.

#### Results

*1. Again, appropriate headings should be provided. Check the grammar*.

**Response:** Appropriate headings have been added to the Results section, and a grammar check has been completed.

*2. Provide a more simplified and summarized Results section. For instance, “In this study, we enrolled 240 respondents, with a mean age....”*

**Response:** The Results section has been simplified and summarized for clarity, including the specific example provided.

*3. Table 1 is very confusing, especially the frequency and percentage columns. Clearly provide both the frequencies and percentages*.

**Response:** Table 1 has been revised to clearly present both frequencies and percentages for better understanding.

*4. Add a key for Figure 2 to give better representation or just integrate the data represented into the text*.

**Response:** A key has been added to Figure 2 for clarity, and relevant data have been integrated into the text for additional context.

#### Discussion

*1. Restate the objective at the start*.

**Response:** The objective of the study has been restated at the beginning of the Discussion section.

*2. Provide a concise summary of key findings*.

**Response:** A concise summary of the key findings has been included at the beginning of the Discussion section.

*3. Thoroughly discuss the implications of the factors found to be significantly associated with hypertension*.

**Response:** A thorough discussion of the implications of the significant factors associated with hypertension has been added to the Discussion section.
